# Epigenetic mediation may explain intergenerational associations between maternal obesogenic lifestyle and children’s birth weight: findings from the NorthPop prospective birth cohort

**DOI:** 10.1186/s13148-025-02001-z

**Published:** 2025-10-29

**Authors:** Kushan De Silva, Richard Lundberg-Ulfsdotter, Stina Bodén, Marie-Therese Vinnars, Patrik Ryden, Christina E. West, Magnus Domellöf, Sophia Harlid

**Affiliations:** 1 Department of Diagnostics and Intervention, Oncology, Umeå University, 901 87 Umeå, Sweden; 2 Helmholtz Institute for Metabolic, Obesity, and Vascular Research, Helmholtz Munich, 04103 Leipzig, Germany; 3 Department of Clinical Sciences, Pediatrics, Umeå University, 901 85 Umeå, Sweden; 4 Department of Clinical Sciences, Obstetrics and Gynecology, Umeå University, 901 85 Umeå, Sweden; 5 Department of Mathematics and Mathematical Statistics, Umeå University, 901 85 Umeå, Sweden

**Keywords:** Birth weight, Classical mediation, Epigenetics, High-dimensional mediation, Intergenerational obesity, Maternal lifestyle

## Abstract

**Background:**

Epigenetic alterations during fetal development have been proposed as key factors explaining associations between maternal lifestyle during pregnancy and later health outcomes in the offspring, pertaining to the developmental origin of health and disease hypothesis.

**Objectives:**

To assess the association of maternal lifestyle with offsprings’ birth weight and underlying epigenetic mediatory mechanisms in the NorthPop prospective birth cohort.

**Methods:**

A three-step analytic pipeline was applied. In 722 mother–child pairs, overall associations between ten maternal lifestyle factors and the offspring’s standardized birth weight were first evaluated by multiple linear regression. Three high-dimensional mediation methods, based on sure independence screening and penalized regression, were then applied on the beta methylation matrix to identify candidate CpG mediators in cord blood driving the significant overall associations. Finally, robust and ordinary least squares (OLS) regression-based classical mediation methods were used with candidate CpG probes to assess single- and multiple (parallel and serial)-mediator models on a low-dimensional space.

**Results:**

Gestational weight gain (GWG) (β-adj = 0.03; *p* = 2 × 10^–5^) and maternal BMI at the beginning of pregnancy (β-adj = 0.036; *p* = 1 × 10^–4^) were significantly associated with the offspring’s standardized birth weight. High-dimensional mediation analyses identified pooled sets of four (cg19242268 [*TCEA2*]; cg08461903 [N/A]; cg14798382 [*CHERP/C19orf44*] and cg21516291 [*SLC35C2*]) and five (cg17040807 [*CYGB*]; cg19242268 [*TCEA2*]; cg26552621 [*CIRBP*]; cg04457572 [*CDH23*] and cg06457011 [*PLCG1*]) candidate CpG mediators related to GWG and BMI at the beginning of pregnancy, respectively. For both exposures, classical mediation analyses revealed a range of significant single- and multiple (both serial and parallel)-mediator models via both robust and OLS regression based approaches. These indicated the likely presence of individual, causally linked multiple, and causally independent multiple mediatory pathways underlying the two significant overall associations.

**Conclusions:**

Our findings support the hypothesis that neonatal health effects related to maternal lifestyle may be partly mediated by epigenetic alterations. Findings also suggest the possible involvement of multiple DNA methylation sites via various mediatory pathways.

**Supplementary Information:**

The online version contains supplementary material available at 10.1186/s13148-025-02001-z.

## Introduction

Epidemiological studies that lend support for the impact of maternal lifestyle on offspring’s life course health are mounting [1–3]. Historical cohorts have indicated intergenerational transmission of adverse health outcomes from pregnant women exposed to extreme conditions such as famines [4, 5]. Current epidemiological evidence suggests that a broad spectrum of maternal lifestyle factors including diet, sedentary behavior, smoking, alcohol consumption, and obesity may have long lasting effects on the offspring’s health [1–3, 6, 7]. For example, maternal smoking during pregnancy may influence fetal development through mechanisms including in utero hypoxia, nicotine-induced uteroplacental blood flow diminution, and placental toxicity [8] while maternal obesity could increase the risk of childhood obesity and overall cardiometabolic health [1–5]. Maternal diet during pregnancy likely affects nutrient availability to the fetus, whereas regular physical activity during pregnancy may optimize maternal health through mechanisms such as blood glucose homeostasis, healthy weight management, and enhanced cardiovascular fitness, leading to improved fetoplacental circulation and reduced risk of preterm birth [6, 7].

Birth weight is a multifaceted indicator of neonatal health reflecting the prenatal environment, nutritional status, fetal growth, and potential risks for both immediate and long-term health outcomes [9, 10]. Low birth weight is known to increase perinatal morbidity and mortality and is associated to poor cardiometabolic health in adulthood [11]. On the other hand, higher birth weights have been linked to elevated risks of obesity and type 2 diabetes later in life [12]. Notably, maternal behaviors such as diet, smoking, stress, and physical activity have also been associated with offspring’s birth weight [9]. It should be noted that in addition to maternal lifestyle, other factors such as maternal genetics [13] and paternal lifestyle [14] are also likely to contribute to later childhood obesity. While DNA methylation is so far the most thoroughly studied mechanism linking lifestyle and exposures during fetal life to later phenotypes, other epigenetic alterations such as histone modifications and non-coding RNA-associated gene silencing are likely to be of equal, or maybe even higher importance. Future efforts to decouple such mechanisms will likely aid our understanding of the generational effects of modifiable exposures in pregnancy [15].

Although mechanisms are still poorly understood, the link between maternal lifestyle and offsprings’ health outcomes is thought to be, at least partly, driven by developmental programming mediated through epigenetic modifications [16]. The concept of developmental programming is underpinned by heightened sensitivity of the developing fetus and the intrauterine environment to external stressors. Maternal metabolic disruptions may induce sustained genetic, phenotypic, and physiologic adaptations in the developing fetus, leading to lasting effects on its future health postnatally [17]. Epigenetic modifications, which entail the modulation of gene expression without altering the original DNA sequence, encompass multiple mechanisms including, histone acetylation, RNA modifications and DNA methylation. In epidemiological studies, DNA methylation is the mechanism that has been most thoroughly studied as it can be readily assessed at a large scale and previous work has supported the theory of epigenetics providing a modifiable link between maternal lifestyle and childhood health risks. One example includes a lifestyle intervention in pregnant women with obesity which was found to impact cord blood DNA methylation, which also associated to body composition in the offspring [18]. The primary aim of the current study was therefore to assess associations between maternal lifestyle and offsprings’ birth weight and evaluate underlying epigenetic mediatory mechanisms. The rationale was that a stepwise approach would be highly suited to this, and we proceeded with the following analytical pipeline: First, investigate which maternal exposures are linked to offspring birthweight, and which covariates are relevant for these associations. Next, assess the CpGs that act as mediators of these associations. Last, examine the specific roles of these mediatory CpGs in the association between maternal exposures and children’s birthweight.

## Materials and methods

### Study population

The NorthPop Birth Cohort Study (NorthPop) is an ongoing population-based, prospective birth cohort conducted in Västerbotten county, Northern Sweden [19]. It includes an extensive longitudinal database and a biobank. NorthPop aims to include 10,000 pregnant women and follow their children through birth until 7 years of age (https://www.umu.se/en/research/infrastructure/northpop/). With prospectively collected, lifestyle-related information of pregnant women, epigenetic measures in cord blood, and follow-up information of children at birth being available, the NorthPop cohort provides a unique opportunity to assess not only the association between maternal lifestyle and the offspring’s health but also associated putative epigenetic mediatory mechanisms.

### Study sample

A sample of 722 mother–child pairs from the NorthPop cohort, with cord blood DNA methylation measured at birth, were included in this study. Participating mothers were selected based on previous parity (primipara single-birth mothers or multiparous twin or triplet mothers) and sample availability. Eligible pregnant women were recruited during the years 2016–2020, from the University Hospital of Umeå catchment area at the time of their routine ultrasound examination at gestational week 14–24. Informed consent was given by all participating women and their partner. Web-based questionnaires were administered to the participating women at multiple times during and after the pregnancy. The first questionnaire was administered during gestational week 14–24, to collect information on socioeconomic status and medical history. Details on lifestyle during pregnancy including diet, physical activity, and stress, were collected through questionnaires provided at gestational week 26–34. Questions about the woman’s health during pregnancy and the health of the newborn were included in a questionnaire sent four months postpartum. Information on maternal education level and country of birth was available to be used as proxy measures of participants’ socioeconomic status.

### Exposures and outcome

Ten maternal lifestyle-related exposures were originally included in the study, with details provided in Supplementary material 1. These comprised physical activity, stress, six different diet-related exposures, gestational weight gain (GWG) and body mass index (BMI) at the beginning of pregnancy (Table 1). The outcome, birth weight, was obtained from The Swedish Pregnancy Register [20] and standardized using the latest published intrauterine growth reference ranges for estimated fetal weight applicable to Sweden [21].

**Table 1:** General characteristics of maternal- and offspring samples in the NorthPop prospective birth cohort analyzed in the present study

Maternal cohort		Offspring cohort	
Characteristic^a^	*N* = 702	Characteristic^a^	*N* = 722
Maternal age (years)^b^	29.9 (4.1)	Birth weight (grams)	3449 (526.2)
Previous parity		Standardized birth weight (z-score)	− 0.42 (0.04)
0	682	Birth length (cm)	50.0 (2.3)
> = 1	20	Gestational age at birth (weeks)	39.8 (1.7)
Physical activity^c,d^	9.4 (4.1)	Apgar	
Less, *n*	434	1-min score	8.4 (1.4)
More, *n*	227	5-min score	9.0 (0.9)
Missing	41	10-min score	9.4 (0.7)
Stress^e,f^	1.9 (2.4)	Missing	
No, *n*	515	Sex, *n*	
Yes, *n*	168	Female	334
Missing, *n*	19	Male	388
DDS^g^	20.7 (5.3)	Delivery Mode, *n*	
DDS-eadj^h^	21.8 (4.7)	Vaginal	610
DII^i^	− 1.2 (1.9)	Cesarean	112
MDS^j^	4.5 (1.7)	Year of birth, *n*	
HNFI^k^	2.5 (1.4)	2016	41
Total energy intake (kcal/d)	2275 (777)	2017	183
Dietary CO_2_ e/DCP	1560.9 (1023.7)	2018	240
GWG^l^(kg)	15.2 (5.6)	2019	180
BMI^m^ (kg/m^2^)	24.5 (4.1)	2020	78
< 18, *n*	5		
18–24.9, *n*	439		
> = 25, *n*	238		
Missing	20		
Smoking^n^, *n*			
No	681		
Yes	2		
Missing	19		
Country of birth^o^, *n*			
Sweden	630		
Other	62		
Missing	10		
Educational level^o^, *n*			
9 year primary school	11		
Upper secondary education	164		
University or university college	516		
Missing	11		

^a^Reported as mean (SD) unless otherwise specified

^b^Mother’s age at delivery

^c^Validated questionnaire-based index

^d^Score < 11 is ‘less’, score ≥ 11 is ‘more

^e^Based on the General Health Questionnaire—12 items (GHQ-12)

^f^Score ≥ 3 is ‘Yes’, score < 3 is ‘No’

^g^Based on 40 food items

^h^Based on 40 food items and adjusted for total energy intake

^i^Based on 30 of the 45 food items used in the original study

^j^An adapted version based on 8 food items

^k^Based on 6 food groups typically consumed in Nordic countries

^l^Self-reported at 4 months postpartum, in kg

^m^Calculated at the beginning of pregnancy

^n^Self-reported at gestational age 26 weeks

^o^Self-reported at gestational age 14–24 weeks

*BMI* body mass index, *DDS* diet diversity score, *Dietary CO2 e/DCP* dietary CO2 emission/dietary carbon footprint, *DII* diet inflammatory index, *GDM* gestational diabetes mellitus, *GWG* gestational weight gain, *HNFI* healthy Nordi food index, *IQR* inter-quartile range, *MDS* mediterranean diet score, *NA* number of missing data, *SD* standard deviation

### DNA methylation data

Cord blood buffy coat DNA samples from the children were bisulphite treated and analyzed for methylation using the Infinium MethylationEPIC BeadChip (Illumina) 850 k v1.0. DNA quality control, pre-processing, processing, and output data quality control were performed at the SNP&SEQ Technology Platform, Uppsala, Sweden, part of the National Genomics Infrastructure (NGI) Sweden and Science for Life Laboratory.

**Fig. 1:**
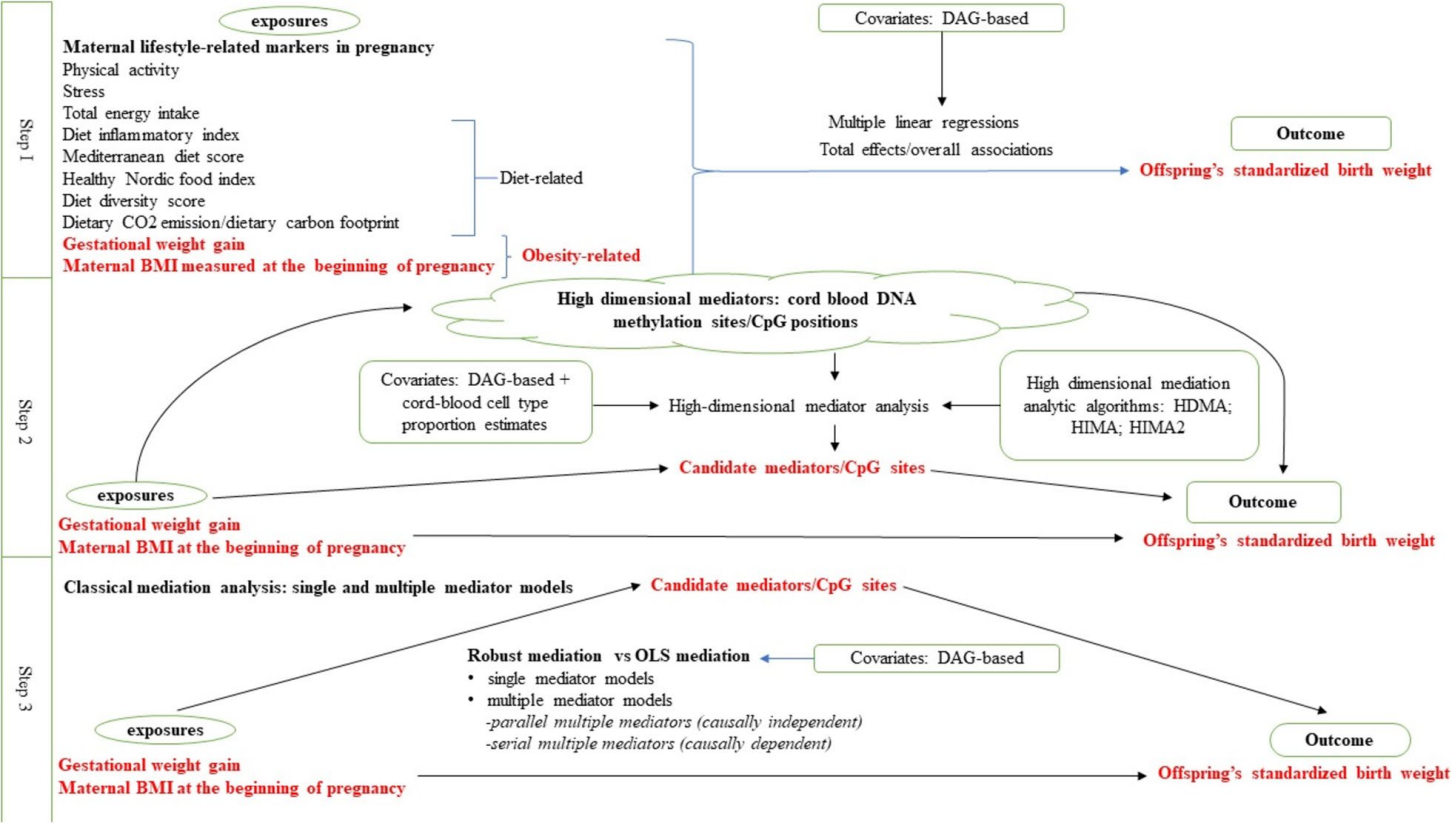
Study workflow

The methodological workflow consisted of three steps as outlined below and presented in Fig. 1.

*Step 1* We applied multiple linear regression to assess overall associations between ten maternal lifestyle-related exposures and offsprings’ standardized birth weight. Directed acyclic graphs (DAGs) were drawn a priori to determine covariates to be included in the analysis of each exposure-outcome association, using the “dagitty” R package [22]. Based on DAGs, the multiple regression modelling the association between maternal BMI at the beginning of pregnancy and standardized birth weight was adjusted for maternal age, maternal education, maternal country of birth, and maternal smoking during pregnancy. The remaining overall association analyses were adjusted for maternal age, maternal BMI at the beginning of pregnancy, maternal education, maternal country of birth, and maternal smoking during pregnancy. Missing data were excluded from the multiple regression analyses. The maternal exposures significantly associated with offsprings’ standardized birth weight as per Step 1, were the focus in subsequent downstream analyses.

As a supplementary analysis, we also assessed maternal BMI-GWG correlations and associations of maternal BMI and GWG with z-birthweight of children in the entire cohort as well as sub-cohorts of obesity, obesity + overweight, overweight, and normal weight.

*Step 2* An account of the methylation data processing and the analytic pipeline is provided in Supplementary material 2. A DNA methylation matrix with beta values produced by the processing pipeline detailed in Supplementary material 2 was used for high-dimensional mediation analyses in Step 2. We applied three high-dimensional mediation methods amenable for DNA methylation data to identify candidate CpG mediators that drive the significant overall associations observed in Step 1. These methods represent recent developments in epigenetic mediation analysis which strive to overcome high-dimensionality by a two-step procedure. Briefly, the initial sure independence screening (SIS) step is followed by a subsequent variable selection step to further reduce dimensions. The ultimate statistical testing is performed on a low-dimensional feature space which has both survived SIS (in step i) and filtering by the subsequent feature selection step (in step ii) to determine significant mediators [23]. All three methods entail some form of penalized regression to estimate mediator-specific contributions [23]. Of these, the “HDMA” [24] and “HIMA” [25] methods were deployed using the “HDMED” R package [23] while the “HIMA2” [26] method was deployed through the “HIMA” R package [25].

In order for the paper to be self-explanatory, we describe the three high-dimensional mediation methods used, including their methodological similarities and differences, with further statistical information available in Supplementary material 3.

SIS: This is a method to address high-dimensionality challenge by first screening the variables based on their marginal correlation with the response variable. Variables are ranked based on their marginal correlations and a subset of the top-ranked variables based on a specified threshold is selected for further analysis. SIS reduces the dimensionality from a high amount to a moderate size, where it is typically chosen to be less than the sample size. The key feature of SIS is its "sure screening" property, meaning that it is designed to retain all the important variables with high probability. This is crucial for ensuring that the subsequent variable selection process focuses on the most relevant features. Methodological information of SIS has been previously published [27].

1. HDMA: In the first feature reduction step, HDMA conducts SIS to determine the number of mediators that are most associated with the outcome (in case of a continuous response variable) or the exposure (in case of a categorical response variable). This initial screening selects features based on *p*-values from linear regression. In the second step of feature reduction, the outcome model is fitted for the remaining mediators using de-sparsified/de-biased LASSO. Next, mediator models are fitted using linear regression among those mediators that have both survived SIS (in step i) and been identified by de-biased LASSO (in step ii), obtaining *p*-values for mediation contributions by taking the maximum of α_a_ (coefficient estimate of the exposure – > mediator association) and β_m_ (coefficient estimate of the mediator – > outcome association adjusted for exposure) *p*-values. Mediation contributions of individual mediators are summed up to estimate the global indirect effect while the direct effect is equivalent to the difference between the total effect and the global indirect effect. Details of the HDMA method have been published elsewhere [24].
2. HIMA: The first step in HIMA is identical to HDMA i.e., SIS to choose mediators that are most associated with the outcome (when the outcome variable is continuous) or the exposure (when the outcome variable is categorical) based on *p*-values from linear regression. Minimax concave penalty (MCP) is used in the second step to fit the outcome model for the remaining mediators. Next, mediator models are fitted using linear regression among those mediators that have both survived SIS (in step i) and been selected by the MCP (in step ii), to determine mediation contributions. Corresponding *p*-values for mediation contributions are estimated in the same way as HDMA, by taking the maximum of α_a_ and β_m_ *p*-values. Multiple testing correction is applied to *p*-values to obtain the ultimate set of statistically significant mediators. The global indirect effect and the direct effect are also enumerated similarly to the HDMA method. The HIMA method is detailed elsewhere [25]
3. HIMA2: Identical to HDMA in terms of SIS (Step i) and de-biased LASSO (Step ii). The difference is that HIMA 2 applies a less conservative multiple testing correction for the joint significance test *p*-values termed “joint significance mixture” approach contrary to “joint significance uniform” approach used in HIMA, aiming to more efficiently detect active mediators. The HIMA2 methodology is elaborated elsewhere [26].

Given the similarities across the three methods, they are in fact categorized within a single group of “penalized regression to estimate mediator-specific contributions” in a study on high-dimensional epigenetic mediation methods [23]. The primary difference between HDMA and HIMA is the penalty function; HDMA uses more-recently introduced de-sparsified/de-biased LASSO whereas HIMA applies MCP. Compared to HIMA, advantages of HDMA include its ability to fit and test multiple mediators in one regression model and superior handling of correlations between methylation sites due to de-sparsifying strategy. Compared to HIMA, strengths of HIMA2 include more accurate SIS screening taking into account both α_a_ and β_m_, and less conservative false discovery rate (FDR) control.

As customarily performed in previous studies [24, 26, 28, 29], we assessed several high-dimensional mediation methods instead of a single method, as there is no gold standard at present [23, 28]. Notably, this multi-algorithmic approach in previous studies has yielded complementary results, identifying both overlapping CpGs and unique methylation loci [24, 26, 28, 29]. This could be attributed to aforementioned methodological similarities and differences between HDMA, HIMA, and HIMA2.

Covariates determined by DAGs and cord blood cell type proportion estimates were included in all high-dimensional mediation analyses. An FDR adjusted *p*-value threshold < 0.05 was applied to further filter the set of CpG sites selected by each high-dimensional mediation method and determine candidate CpG mediators. Findings from each method were merged to produce the pooled set of candidate CpG mediators. Classical mediation analyses were performed on these candidate CpG mediators in Step 3.

*Step 3* Typically, mediation analysis entails a series of linear regressions whereby indirect effects are enumerated as products of regression coefficients and their statistical significance is determined by a bootstrap test based on ordinary least-squares (OLS) estimates. The OLS-regression-based test is sensitive to deviations from normality assumptions or the presence of outliers hindering empirical testing of mediation mechanisms. In contrast, robust regression-based mediation is resistant to deviations such as outliers or skewed distributions, which utilizes the robust MM-regression estimator instead of the OLS estimator for regression. In order to get a comprehensive understanding of causal mediatory pathways and compare results between the two methods, we assessed epigenetic mediation on a low-dimensional space via both robust-regression based bootstrap method and OLS-regression-based bootstrap method using the ‘robmed’ R package [30]. DNA methylation beta values of the candidate CpG mediators produced by the processing pipeline detailed in Supplementary material 2 was used for classical mediation analyses in Step 3. With respect to each significant overall association identified in Step 1, we analyzed single mediator models, multiple serial mediator models (assuming causal dependence between multiple mediators), and parallel mediator models (assuming causal independence between multiple mediators). All analyses were adjusted for DAG-based covariates. As the number of mediatory pathways combinatorially increase in serial models quickly growing in complexity, the ‘robmed’ package allows only a maximum of three mediators in serial multiple mediation analyses. We determined the three CpG mediators to be included in serial mediation analyses, based on the results from single mediation assessments.

Finally, we searched the CpG mediators identified by the present study on several databases in order to obtain biological insights. These included the MRC-IEU catalog of epigenome-wide association studies (EWAS Catalog) [31] and the EWAS Atlas [32] to uncover any consistent findings reported in previous studies, the EPIGEN MeQTL Database (https://epicmeqtl.kcl.ac.uk/) to examine associated genetic variants, and the eFORGE TF [33] to identify overlapping with known transcription binding-sites to regulate gene expression.

## Results

General characteristics of the maternal—offspring paired cohorts, including maternal exposures and standardized birthweight of offsprings, analyzed in the present study are summarized in Table 1. Body composition of the offspring measured as z-birthweight and birth length are reported (Table 1).

*Step 1* Associations between maternal exposures and offspring’s birthweight.

Results from DAGs analysis that determined the covariates to be included in overall association analyses are presented in Supplementary material 4. In the adjusted linear regression models, two maternal lifestyle markers, gestational weight gain (GWG) and BMI at the beginning of pregnancy, were significantly associated with offspring’s standardized birth weight (β_GWG_ = 0.03; 95% CI 0.02–0.04 and β_BMI_ = 0.036; 95% CI 0.019–0.054) (Table 2).

**Table 2:** Overall associations between maternal lifestyle markers and offspring’s standardized birth weight in the NorthPop prospective birth cohort as per simple- and multiple- linear regression

Maternal lifestyle factor	β-unadj	95% CI of β-unadj	*p*-value	β-adj	95% CI of β-adj	*p*-value
GWG^a^	**0.034**	**0.021 to 0.047**	**6e−07**	**0.031**	**0.021 to 0.042**	**2e−05**
Physical activity (continuous)^a^	− 0.0011	− 0.0187 to 0.0167	0.91	− 0.0009	− 0.0192 to 0.0178	0.92
More physical activity (Ref. = Less)^a^	− 0.026	− 0.179 to 0.127	0.74	0.021	− 0.134 to 0.176	0.79
BMI at the beginning of pregnancy^b^	**0.039**	**0.02 to 0.055**	**3e−05**	**0.036**	**0.019 to 0.054**	**1e−04**
Total energy intake^a^	0.00003	− 0.00007 to 0.00011	0.58	0.00005	− 0.00004 to 0.00014	0.28
Stress (continuous)^a^	0.017	− 0.013 to 0.047	0.27	0.007	− 0.024 to 0.039	0.65
More stress (Ref = Less)^a^	0.027	− 0.138 to 0.193	0.75	− 0.026	− 0.196 to 0.143	0.76
Diet diversity score^a^	0.003	− 0.011 to 0.017	0.69	0.009	− 0.005 to 0.023	0.22
Dietary CO_2_ emission/carbon footprint^a^	0.00001	− 0.00006 to 0.00008	0.69	0.000008	− 0.00006 to 0.00008	0.83
Diet inflammatory index^a^	0.025	− 0.013 to 0.063	0.20	0.007	− 0.032 to 0.046	0.73
Mediterranean diet score^a^	− 0.038	− 0.081 to 0.005	0.09	− 0.017	− 0.062 to 0.027	0.45
Healthy Nordic food index^a^	0.022	− 0.03 to 0.075	0.40	0.042	− 0.011 to 0.096	0.12

^a^Adjusted for maternal age, maternal BMI at the beginning of pregnancy, maternal education, maternal country of birth, maternal smoking during pregnancy

^b^Adjusted for maternal age, maternal education, maternal country of birth, maternal smoking during pregnancy

*BMI* body mass index, *CI* confidence interval, *GWG* gestational weight gain, *β-adj* effect size measured as the multiple linear regression coefficient, *β-unadj* effect size measured as the simple linear regression coefficient

Maternal BMI-GWG correlations and their multivariable associations with children’s z-birthweight are summarized in Supplementary material 5. Maternal BMI at the beginning of pregnancy and GWG were positively correlated, albeit weakly, in the entire cohort and in the normal weight sub-cohort. In obesity, obesity + overweight, and overweight sub-cohorts, the correlations were negative, albeit weak. The inclusion of these maternal exposures did not substantially change their significant associations with children’s z-birthweight, except in the sub-cohort of women with obesity in which only GWG remained significant when both maternal BMI and GWG were included. Both these maternal exposures remained independently associated with z-birthweight in the full cohort and all sub-cohorts except in pregnant women with obesity.

The original EPIC array had 862,452 CpG probes, after the quality control steps detailed in Supplementary material 2 were performed, 755,671 unique CpG probes were retained for interrogation.

*Step 2* High-dimensional epigenetic mediation analysis.

The HDMA method identified 21 CpG sites mediating the association between GWG and offspring’s standardized birth weight, four of which (cg19242268; cg08461903; cg14798382; cg21516291) passed an FDR adjusted threshold of 0.05 and were selected as candidate CpG sites for classical causal mediation analysis. The HIMA method derived a set of 24 CpG sites mediating the association between GWG and offspring’s standardized birth weight, 3 of which (cg19242268; cg08461903; cg21516291) passed the FDR-adjusted threshold of 0.05 and were selected as candidate CpG sites for classical causal mediation analysis. Finally, the HIMA2 method also identified 24 CpG sites as mediating the association between GWG and the offsprings’ standardized birth weight, 2 of which (cg19242268; cg08461903) passed the FDR-adjusted threshold of 0.05 and were selected for classical causal mediation analysis. The pooled set of four candidate CpG sites eligible for classical mediation analysis of the association between GWG and the offspring’s standardized birth weight included the same four CpG sites as captured by the HDMA method (cg19242268; cg08461903; cg14798382 and cg21516291) (Table 3; Supplementary material 6).

**Table 3:** Summary of results from high-dimensional mediation analysis including the candidate CpG sites selected as mediators

High-dimensional mediation analytic approach	CpG sites selected by high-dimensional mediation approach after 2-step dimension reduction		Candidate CpG sites selected for classical causal mediation analysis (FDR-adjusted *p* < 0.05)	
	Number	Composition	Number	Composition
Association between GWG and offspring’s z-birth weight				
HDMA	(*n* = 21)	cg04968127; cg14798382; cg21516291; cg27053299; cg10660916; cg14556683; cg10178960; cg16752400; cg09247736; cg18137450; cg07002832; cg02832224; cg05779272; cg19242268; cg16402875; cg15672022; cg13131501; cg04457572; cg01940139; cg08461903; cg00154986	(*n* = 4)	cg19242268; cg08461903; cg14798382; cg21516291
HIMA	(*n* = 24)	cg04968127; cg05349624; cg21516291; cg27053299; cg10660916; cg14556683; cg10178960; cg16752400; cg05304729; cg04751761; cg09247736; cg18137450; cg02832224; cg05779272; cg19242268; cg05560494; cg16402875; cg13131501; cg12804755; cg04457572; cg22247250; cg08461903; cg00154986; cg12145085	(*n* = 3)	cg19242268; cg08461903; cg21516291
HIMA2	(*n* = 24)	cg04968127; cg05349624; cg21516291; cg27053299; cg10660916; cg14556683; cg10178960; cg16752400; cg05304729; cg04751761; cg09247736; cg18137450; cg02832224; cg05779272; cg19242268; cg05560494; cg16402875; cg13131501; cg12804755; cg04457572; cg22247250; cg08461903; cg00154986; cg12145085	(*n* = 2)	cg19242268; cg08461903
Association between maternal BMI and offspring’s z-birth weight				
HDMA	(*n* = 25)	cg04968127; cg21516291; cg23260105; cg00376553; cg17040807; cg21649604; cg16752400; cg25494075; cg08289567; cg18137450; cg02832224; cg05779272; cg19242268; cg26552621; cg18034719; cg09171931; cg13131501; cg03688987; cg04457572; cg06457011; cg14787880; cg01940139; cg08461903; cg00154986; cg12145085	(*n* = 5)	cg17040807; cg19242268; cg26552621; cg04457572; cg06457011
HIMA	(*n* = 24)	cg04968127; cg14798382; cg21516291; cg23260105; cg00376553; cg17040807; cg16752400; cg05304729; cg15482893; cg18137450; cg02832224; cg05779272; cg19242268; cg26552621; cg18034719; cg13131501; cg03688987; cg04457572; cg06457011; cg14787880; cg01940139, cg08461903; cg00154986; cg05632420	(*n* = 5)	cg17040807; cg19242268; cg26552621; cg04457572; cg06457011
HIMA2	(*n* = 24)	cg04968127; cg14798382; cg21516291; cg23260105; cg00376553; cg17040807; cg16752400; cg05304729; cg15482893; cg18137450; cg02832224; cg05779272; cg19242268; cg26552621; cg18034719; cg13131501; cg03688987; cg04457572; cg06457011; cg14787880; cg01940139; cg08461903; cg00154986; cg05632420	–	–

*BMI* body mass index, *GWG* gestational weight gain

The same methods were used to identify CpG sites mediating the association between maternal BMI at the beginning of pregnancy and the offspring’s standardized birth weight. The HIMA, HDMA, and HIMA2 methods yielded 24, 25, and 24 CpG mediatory sites, respectively. Of these, both HDMA and HIMA output comprised the same subset of 5 candidate CpG sites that passed the FDR-adjusted threshold of 0.05 and were selected for classical causal mediation analysis (cg17040807; cg19242268; cg26552621; cg04457572; cg06457011) (Table 3; Supplementary material 6).

*Step 3* Low-dimensional epigenetic mediation analysis.

**Fig. 2:**
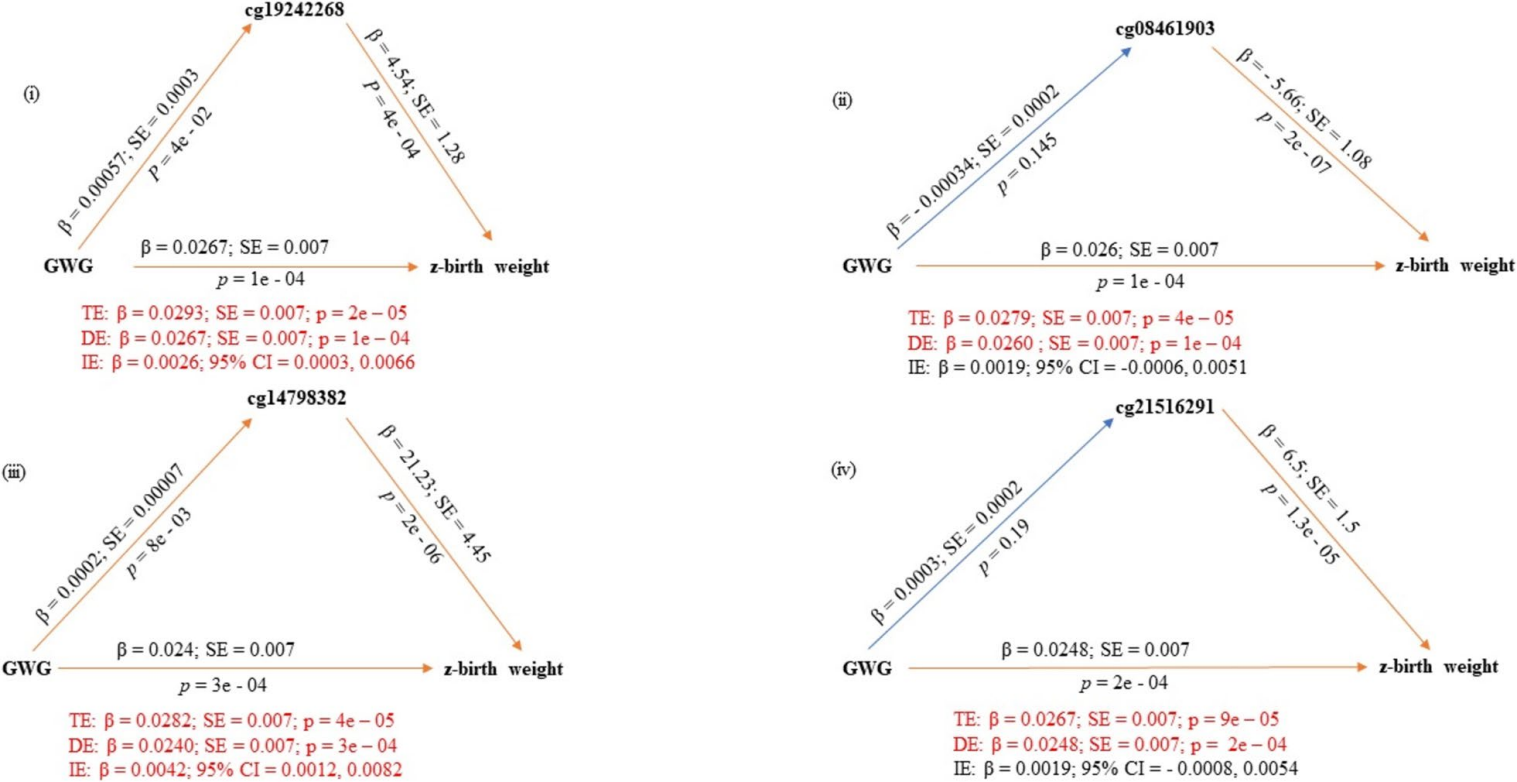
Single mediator models as per the robust bootstrapped approach with candidate CpG sites as mediators of the association between GWG and offspring’s z-birth weight. Significant pathways are drawn in orange while non-significant pathways are drawn in blue. Red text indicates coefficients of significant pathways, their standard errors, *p*-values, and 95% confidence intervals. Black text indicates those values in non-significant pathways. *DE* direct effects, *IE* indirect effects, *TE* total effects

Two robust regression-based single mediator models (cg19242268; cg14798382) (Table 4; Fig. 2) and three OLS regression-based single mediator models (cg19242268; cg14798382; cg08461903) (Supplementary material 7; Supplementary material 8) examining the association between GWG and offspring’s standardized birth weight were significant. All three CpG sites were also identified by the high-dimensional mediation analyses described above and included in multiple mediator models.

**Table 4:** Summary of results from robust mediation analysis with candidate CpG sites selected by high-dimensional mediation analysis

Epigenetic mediation of the association between gestational weight gain and offspring’s standardized birth weight								
Single mediator models—robust bootstrap approach								
CpG	Indirect effect		Direct effect			Total effect		
	Estimate	95% CI	Estimate	SE	*p*-value	Estimate	SE	*p*-value
cg19242268	0.0026	0.0003, 0.0066	0.0267	0.007	0.0001	0.0293	0.007	0.00002
cg08461903	0.0019	− 0.0006, 0.0051	0.0260	0.007	0.0001	0.0279	0.007	0.00004
cg14798382	0.0042	0.0012, 0.0082	0.0240	0.007	0.0003	0.0282	0.007	0.00004
cg21516291	0.0019	− 0.0008, 0.0054	0.0248	0.007	0.0002	0.0267	0.007	0.00009

Serial multiple mediator model—robust bootstrap approach								
Pathway	Indirect effects		Direct effect			Total effect		
	Estimate	95% CI	Estimate	SE	*p*-value	Estimate	SE	*p*-value
Total indirect	0.00703	0.00267, 0.01218	0.01898	0.007	0.005	0.02601	0.007	0.0002
Indirect1	0.00223	0.00025, 0.00578	**(Indirect1: GWG—> cg19242268—> z-birth weight)**					
Indirect2	0.00167	− 0.00065, 0.00462	(Indirect2: GWG—> cg08461903—> z-birth weight)					
Indirect3	0.00267	0.00017, 0.00624	**(Indirect3: GWG—> cg14798382—> z-birth weight)**					
Indirect4	0.00010	− 0.00006, 0.00055	(Indirect4: GWG—> cg19242268—> cg08461903—> z-birth weight)					
Indirect5	0.00014	− 0.00002, 0.00062	(Indirect5: GWG—> cg19242268—> cg14798382—> z-birth weight)					
Indirect6	0.00021	− 0.00005, 0.00071	(Indirect6: GWG—> cg08461903—> cg14798382—> z-birth weight)					
Indirect7	0.00001	− 0.00001, 0.00009	(Indirect7: GWG—> cg19242268—> cg08461903—> cg14798382—> z-birth weight)					

Parallel multiple mediator model—robust bootstrap approach								
Pathway	Indirect effects		Direct effect			Total effect		
	Estimate	95% CI	Estimate	SE	*p*-value	Estimate	SE	*p*-value
Total indirect	0.0075	0.0032, 0.0127	0.0190	0.007	0.005	0.0265	0.007	0.0001
cg19242268	0.0022	0.0002, 0.0058						
cg08461903	0.0018	− 0.0005, 0.0048						
cg14798382	0.0035	0.0010, 0.0071						

Epigenetic mediation of the association between BMI at the beginning of pregnancy and offspring’s standardized birth weight								
Single mediator models—robust bootstrap approach								
CpG	Indirect effect		Direct effect			Total effect		
	Estimate	95% CI	Estimate	SE	*p*-value	Estimate	SE	*p*-value
cg17040807	0.0033	− 0.0001, 0.0085	0.0308	0.009	0.001	0.0341	0.009	0.0003
cg19242268	0.0037	0.0004, 0.0084	0.0310	0.009	0.0006	0.0347	0.009	0.0001
cg26552621	0.0051	0.0012, 0.0107	0.0313	0.009	0.001	0.0364	0.009	0.0001
cg04457572	0.0041	0.0006, 0.0097	0.0327	0.009	0.0002	0.0368	0.009	0.00007
cg06457011	0.0030	− 0.0006, 0.0078	0.0318	0.009	0.0006	0.0348	0.009	0.0002

Serial multiple mediator model—robust bootstrap approach								
Pathway	Indirect effects		Direct effect			Total effect		
	Estimate	95% CI	Estimate	SE	*p*-value	Estimate	SE	*p*-value
Total indirect	0.009124	0.003671, 0.016250	0.026174	0.009	0.003	0.035298	0.009	0.0001
Indirect1	0.002940	0.000405, 0.007337	**(Indirect1: BMI—> cg19242268—> z-birth weight)**					
Indirect2	0.002378	0.000245, 0.006358	**(Indirect2: BMI—> cg26552621—> z-birth weight)**					
Indirect3	0.002302	− 0.000221, 0.006550	(Indirect3: BMI—> cg04457572—> z-birth weight)					
Indirect4	0.000439	0.000047, 0.001313	**(Indirect4: BMI—> cg19242268—> cg26552621—> z-birth weight)**					
Indirect5	− 0.000005	− 0.000317, 0.000271	(Indirect5: BMI—> cg19242268—> cg04457572—> z-birth weight)					
Indirect6	0.000903	0.000146, 0.002294	**(Indirect6: BMI—> cg26552621—> cg04457572—> z-birth weight)**					
Indirect7	0.000167	0.000024, 0.000482	**(Indirect7: BMI—> cg19242268—> cg26552621—> cg04457572—> z-birth weight)**					

Parallel multiple mediator model—robust bootstrap approach								
Pathway	Indirect effects		Direct effect			Total effect		
	Estimate	95% CI	Estimate	SE	*p*-value	Estimate	SE	*p*-value
Total indirect	0.0102	0.0041, 0.0177	0.0232	0.009	0.009	0.0334	0.009	0.0002
cg17040807	0.0015	− 0.00009, 0.0051						
cg19242268	0.0027	0.0004, 0.0068						
cg26552621	0.0015	− 0.0003, 0.0052						
cg04457572	0.0026	0.0004, 0.0072						
cg06457011	0.0019	− 0.0001, 0.0061

**Fig. 3:**
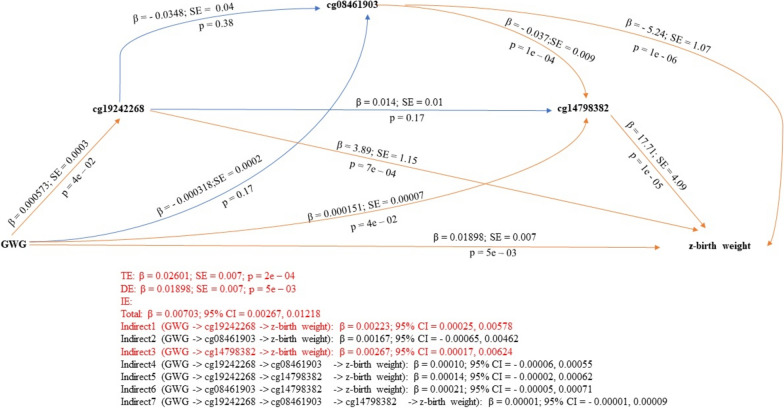
Serial multiple mediator model as per the robust bootstrapped approach with candidate CpG sites as mediators of the association between GWG and offspring’s z-birth weight. Significant pathways are drawn in orange while non-significant pathways are drawn in blue. Red text indicates coefficients of significant pathways, their standard errors, *p*-values, and 95% confidence intervals. Black text indicates those values in non-significant pathways. *DE* direct effects, *IE* indirect effects, *TE* total effects

When examining serial multiple mediation in relation to the GWG-z-birthweight association, we identified several significant indirect pathways, two in robust regression-based serial models (cg19242268; cg14798382) (Table 4; Fig. 3) and five in OLS regression-based serial models (cg19242268; cg08461903; cg14798382; GWG → cg19242268 → cg14798382 → z-birth weight; GWG → cg08461903 → cg14798382 → z-birth weight) (Supplementary material 7; Supplementary material 8).

**Fig. 4:**
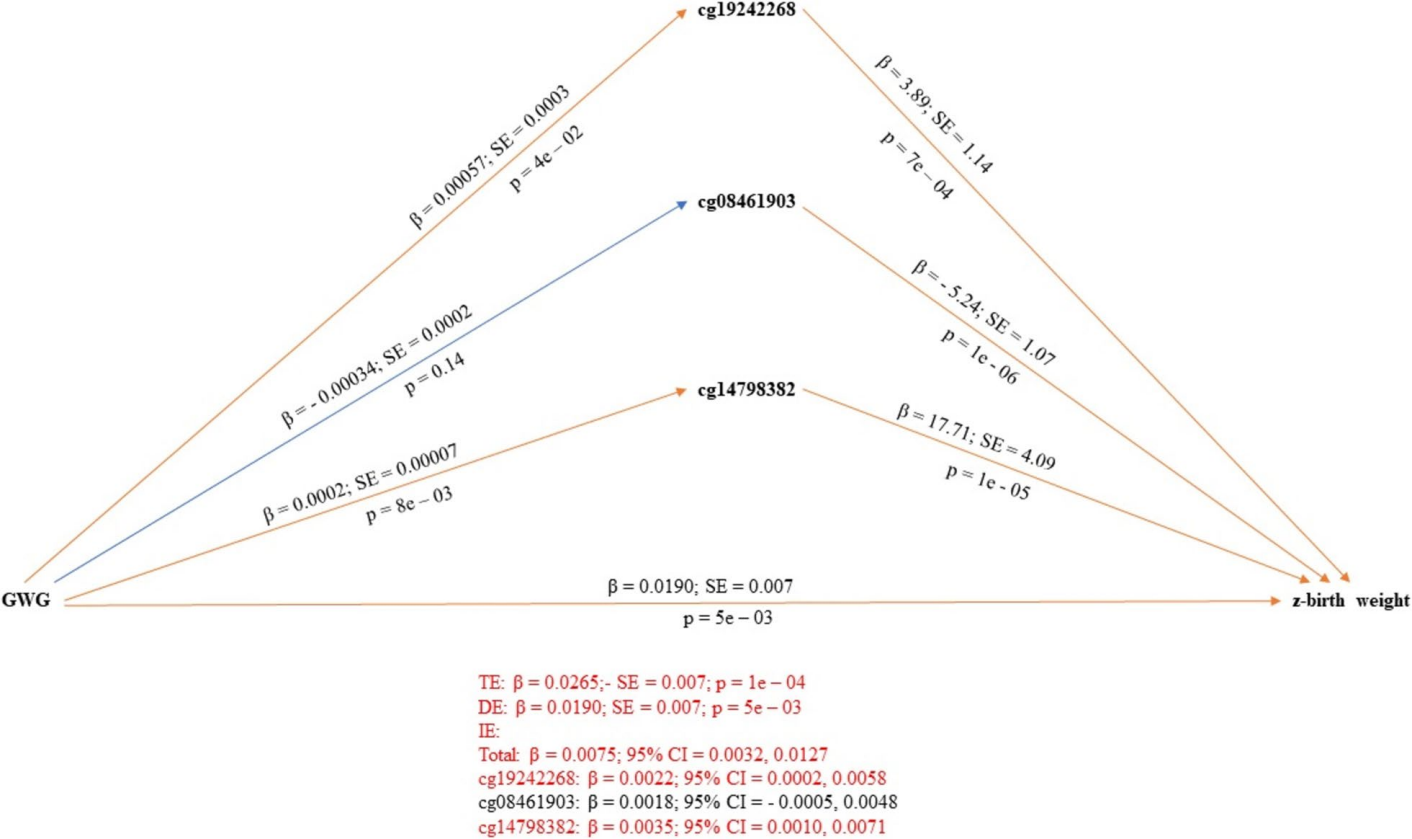
Parallel multiple mediator model as per the robust bootstrapped approach with candidate CpG sites as mediators of the association between GWG and offspring’s z-birth weight. Significant pathways are drawn in orange while non-significant pathways are drawn in blue. Red text indicates coefficients of significant pathways, their standard errors, *p*-values, and 95% confidence intervals. Black text indicates those values in non-significant pathways. *DE* direct effects, *IE* indirect effects, *TE* total effects

Robust parallel multiple mediation of GWG's association with offspring’s standardized birth weight revealed two significant indirect pathways (cg19242268 and cg14798382) (Table 4; Fig. 4), whereas OLS parallel multiple mediation of the same association found three indirect pathways (cg19242268; cg08461903; cg14798382) (Supplementary material 7; Supplementary material 8).

Three robust single mediator models (cg19242268: cg26552621; cg04457572) (Table 4) and all five OLS single mediator models (cg17040807; cg19242268; cg26552621; cg04457572; cg06457011) examining the association between maternal BMI and offspring’s standardized birth weight were significant (Supplementary material 7; Supplementary material 8). The three significant CpG sites in robust single mediator models were included in serial multiple mediation analysis.

In relation to maternal BMI-children’s z-birthweight association, we observed several significant indirect pathways as per serial multiple mediation, five robust pathways (cg19242268; cg26552621; BMI → cg19242268 → cg26552621 → z-birth weight; BMI → cg26552621 → cg04457572 → z-birth weight; BMI → cg19242268 → cg26552621 → cg04457572 → z-birth weight) (Table 4) and six OLS pathways (cg19242268; cg26552621; cg04457572; BMI → cg19242268 → cg26552621 → z-birth weight; BMI → cg26552621 → cg04457572 → z-birth weight; BMI → cg19242268 → cg26552621 → cg04457572 → z-birth weight) (Supplementary material 7; Supplementary material 8).

In contrast, parallel robust multiple mediation revealed two significant indirect pathways (cg19242268; cg04457572) (Table 4) while OLS multiple parallel mediation found three significant pathways (cg19242268; cg04457572; cg06457011) (Supplementary material 7; Supplementary material 8) for the association between maternal BMI and offspring’s standardized birthweight.

In total, eight CpG sites were selected as potential mediators of associations between GWG or pregnancy BMI and birth weight (Table 5). Previous studies on the association of candidate CpG sites with markers of obesity found on the EWAS Catalog are summarized in Supplementary material 9. A single candidate CpG site, namely, cg19242268 was found to mediate both significant overall associations, emerging significant in all single- and multiple- mediator models.

**Table 5:** Annotated details of the CpG sites selected as mediators of the association between gestational weight gain/maternal BMI at the beginning of pregnancy and offspring’s standardized birth weight

CpG site	Exposure(s)	CHR	Position	Gene	Gene region	Relation to island
cg14798382	GWG	chr19	16,629,806	CHERP; C19orf44	3'UTR	N_Shore
cg19242268	GWG & BMI	chr20	62,688,573	TCEA2	1st Exon; 5'UTR	Island
cg21516291	GWG	chr20	44,979,100	SLC35C2	Body	OpenSea
cg08461903	GWG	chr21	45,884,825	–	–	S_Shore
cg17040807	BMI	chr17	74,533,282	CYGB	Body	Island
cg26552621	BMI	chr19	1,271,019	C19orf23; CIRBP	TSS 1500; Body	S_Shore
cg04457572	BMI	chr10	73,303,234	CDH23	Body	OpenSea
cg06457011	BMI	chr20	39,767,490	PLCG1	Body	S_Shore

*BMI* body mass index, *GWG* gestational weight gain, *CHR* chromosome

We present findings on the direction of effects, correlations, and associations as reported on EWAS Catalog and EWAS Atlas from previous studies in relation to methylation markers and exposures (GWG, BMI) or related traits and outcome (birthweight) in Supplementary material 10.

## Discussion

We identified eight potential CpG mediators that could be mapped to genes with obesogenic potential. Seven of these mediated associations between either GWG or pregnancy BMI and birth weight, whereas one, cg19242268, stood out as a potential mediator in all models. Cg19242268 is positioned in a CpG island situated in the first exon of one isoform of the gene *TCEA2* (Transcription Elongation Factor A2), the protein of which is involved in transcriptional regulation and mainly expressed in the testis and brain. Interestingly, at least one previous study identified a differentially methylated region (DMR) in cord blood associated with birthweight that overlapped with the promoter of *TCEA2* and another gene (*RP13-152O15.5*) [34], lending further support for its involvement as an important epigenetic mediator of weight.

Another CpG of interest was cg14798382 which mapped to the *CHERP* (calcium homeostasis ER protein) gene, previously shown to be involved in cellular growth and proliferation through the regulation of calcium homeostasis [35]. In another study which aimed to identify genes associated with nonalcoholic fatty liver disease, *CHERP* was shown to be strongly downregulated in afflicted individuals [36], but not much is known about its potential involvement in disease development so far.

Other CpGs of interest were situated in the genes *SLC35C2* (cg21516291), *CYGB* (cg17040807), *CIRBP* (cg26552621) and *PCLG1* (cg06457011). The *SCL35C2* gene regulates glycosylation—an essential post-translational modification process important for multiple biological processes, including embryonic development [37]. *CYGB* is essential for regulation of adipogenesis, inflammation, blood pressure, and oxidative stress response [38, 39], *CIRBP* is involved in regulation of glucose metabolism, adipose tissue function, and inflammation [40, 41] and finally, *PLCG1* is involved in insulin signaling, leptin signaling, and the regulation of adipose tissue functions [42]. Taken together, mediatory CpG sites found by the present study are situated close to multiple genes capable of elevating an individual’s obesogenic risk through diverse functional pathways.

Furthermore, consistent findings from existing literature on the EWAS Catalog [31] add to the biologically plausibility. A multi-ancestry meta-analysis of epigenome-wide association studies revealed that three mediators identified by the present study (cg19242268 in *TCEA2*, cg21516291 in *SLC35C2*, and cg17040807 in *CYGB*) are all previously identified DNA methylation markers of birth weight [43]. Meanwhile, a previous EWAS reported that seven of the methylation mediators revealed by the current study (cg19242268, cg21516291, cg26552621, cg04457572, cg06457011, cg14798382, cg08461903) associate with childhood growth trajectories from birth to late adolescence [44]. Moreover, two mediators in the present study (cg21516291 and cg04457572) were identified as epigenetic markers of incident type 2 diabetes by another EWAS [45]. These findings from previous EWAS studies bolster the credible link between the CpG mediators identified through the present analysis and obesity phenotypes.

Interestingly, an integrated methylome- and phenome-wide assessment of the circulating proteome revealed the associations of cg26552621 in *CIRB2* (identified as an epigenetic mediator in the present study) with obesogenic NOG protein levels and cg04457572 in *CDH23* (another epigenetic mediator in the present study) with ADIPOQ protein/adiponectin levels which regulate fat metabolism and insulin sensitivity [46]. Moreover, an integrative cross-omics analysis of DNA methylation sites of glucose and insulin homeostasis found that a third epigenetic mediator of the current study, cg06457011 in *PLCG1* was associated with fasting insulin while differential methylation explained at least 16.9% of the association between obesity and insulin [47]. Some of these obesity-associated methylation signatures have been robustly replicated across cohorts [43]. The discovery of cg19242268 as a mediator with respect to both maternal BMI and GWG is intriguing. Notably, cg19242268 is also associated with birthweight as per previous EWASs [34]. Our findings may allude to an important epigenetic signal and further investigations are warranted.

As per EWAS Atlas [32], two of the CpG mediators (cg19242268 in the *TCEA2* gene and cg21516291 in the *SLC35C2* gene) are hypermethylated in relation to birthweight. In tandem, EWAS Catalog [31] revealed both are positively associated with birthweight (Supplementary material 10). As revealed by high-dimensional mediation analyses, mediation pathways of cg14798382 (GWG → cg14798382 and cg14798382 → z-birthweight) were positive (Supplementary material 6), indicating potential hypermethylation and downregulation of CHERP*/C19 orf44*. However, we note the presence of inconsistencies in the direction and correlation/association of methylation for the same phenotype reported by different EWAS studies. Therefore, caution is warranted when interpreting findings related to hypermethylation or hypomethylation, and further research is needed to confirm their effects and their directional impact.

Previous large-scale observational epidemiological studies have linked maternal obesity with large-for-gestational-age offspring [48, 49] and suggested a potential causal relationship [50] and several of the methylation sites identified in our study have been connected to birth weight, obesity, and diabetes. For instance, as per EWAS Catalog [31], a previous EWAS revealed that cg19242268 and cg21516291 are positively associated with birthweight whereas cg17040807 is negatively associated with birthweight [42] (Supplementary material 10). However, information on mediatory pathways have been lacking. Using both robust regression-based and OLS regression-based multiple mediation analyses we have been able to unravel potentially causally linked and causally independent mediatory pathways involving multiple methylation sites. We thus provide suggestive evidence that the methylation sites may exert their mediatory effects individually as well as concomitantly via complex pathways. These statistically intuitive findings together with existing literature shed light on the complex nature of epigenetic mediation that may underlie later health effects in the offspring highlighting epigenetic mediation as a likely mechanism contributing to intergenerational obesity.

The present study adopted a comprehensive analytic pipeline. It comprised the application of multiple topical high-dimensional mediation methods followed by both OLS- and robust classical mediation analyses and the evaluation of single as well as multiple mediatory models. High-dimensional mediation methods are especially amenable to DNA methylation data as revealed by previous birth cohort studies yielding novel findings [28, 29, 51].

According to our knowledge, this is the first and the largest prospective birth cohort study in a Northern European setting to unravel the epigenetically mediated association of two obesogenic maternal lifestyle markers in pregnancy with children’s birth weight. A previous birth cohort study provided some evidence of epigenetics being involved in the intergenerational risk of obesity, however, their study population consisted of a predominantly urban, low-income ethnic minority and results might therefore be difficult to generalize for other populations [52].

Our study also has imitations. Although, our sample is of considerable size compared to most epigenetic studies reported earlier, larger cohorts may be required to gain more robust findings with higher statistical power. The sample size in this study is noticeably large compared to contemporary epigenetic mediation studies [53]. Sample size or statistical power estimation methods for epigenetic mediation assessment are sparse, still nascent with no wide acceptance, and there is a lack of consensus on a single, standard strategy. A recent study revealed that for achieving a statistical power of 80% in causal mediation studies with small effect sizes, 413 samples would be required to determine total indirect effects, when both mediator and outcome are continuous [54]. Noteworthily, most contemporary epigenetic mediation studies have not performed formal sample size/statistical power calculations [53].

The presence of residual confounding may have influenced effect estimates, despite the inclusion of an array of DAG-based covariates. The genetic heritability component including the effect of maternal genetics on children’s obesity is non-trivial [55] and could confound, modulate, or interact with epigenetic effects or independently associate with birthweight. Therefore, epigenetic mediation is unlikely to fully explain the association between maternal obesogenic traits and children’s birthweight, and other mediatory mechanisms are worthy of being explored. For example, several CpG loci in our study (cg19242268; cg08461903; cg06457011) associate with SNPs, alluding to the possible influence of genetic effects on DNA methylation (Supplementary material 10).

Since some of the identified CpGs are associated with smoking, we reconducted all analyses omitting the two smokers in the cohort, which did not change the findings. We also note that all reported analyses were adjusted for maternal smoking in pregnancy. Still, we acknowledge that the cohort is homogeneous in terms of smoking, as there were only two self-reported smokers, making it impossible to assess the potential impact of smoking. Since non-responders to the questionnaire on smoking could be smokers or non-smokers, we did not exclude them in our re-analyses, but a “sensitivity analysis” omitting non-responders as well could have been informative. Exposure to secondhand smoking and thirdhand smoking in non-smoking pregnant women could further confound the associations. Therefore, confounding of the effects by maternal exposure to smoking cannot be entirely ruled out in the present study.

Socioeconomic status of participants was proxied via their education level and the country of birth. We acknowledge that these two covariates may not have fully accounted for socioeconomic-driven confounding and additional information such as income may have been useful. Another limitation is that mothers participating in these studies are more often highly educated and have higher incomes than mothers in the general population, raising generalizability issues [56]. Our cohort was relatively homogeneous with only 11 participants having education lower than upper secondary level and a majority born in Sweden. However, the NorthPop cohort covers a large catchment area representing the whole Västerbotten region and selective participation is unlikely to have been a major issue.

Maternal BMI and GWG were only weakly correlated, and revealed independent associations with children’s birthweight in our cohort, except in pregnant women with obesity. While we did not conduct sub-analyses among pregnant women with obesity, due to the smaller sub-cohort size (*N* = 90), future studies in this direction may provide valuable insight. As methods for multiple exposure-multiple mediator-single outcome models are not widely available, and tend to be overly complex and less intuitive, we did not conduct mediation analyses that simultaneously incorporated both maternal exposures.

Significant results of our study pertain to maternal BMI and GWG only, although ten maternal lifestyle factors were assessed initially. Lack of statistical power to reveal associations may be a possible explanation that other maternal lifestyle factors did not achieve significance. This warrants future analyses on larger samples. The U-shaped relationship of birthweight with health outcomes reported in previous literature [57, 58] might not be apparent in our cohort given the likely under-sampling of underweight pregnant women in the maternal cohort and offsprings at the lower end of the birthweight spectrum. Of note, there were only 5 underweight (< 18 kg/m^2^) participants in the maternal cohort. Hence, associations of the present analysis could have been driven by an overrepresentation of pregnant women with overweight/obesity.

It should be noted that besides maternal genetics and maternal lifestyle, there is mounting evidence that other modifiable and non-modifiable factors also contribute to intergenerational obesity. For example, a recent study provided epidemiologic and functional evidence of paternal contribution to offspring obesity and metabolic risk mediated through changes in sperm, including epigenetic modifications [59]. Modifiable factors such as paternal diet and paternal smoking are also associated with offspring’s birthweight [59–61].

At present, genes associated with CpG mediators in this study are not directly linked to obesity in the same way as genes like *MC4R* or *FTO*. However, epigenetic modifications affecting these genes can play a role in obesity development by influencing how genes are turned on or off in response to environmental factors like maternal obesogenic lifestyle. Our findings highlight the necessity for conducting future studies to unravel their role in intergenerational obesity.

Causal inference cannot be drawn from observational designs and future studies are recommended to validate our findings. Furthermore, despite there being potential connections between birthweight and early childhood BMI trajectories the follow-up time in the current study was too short to study such associations. However, NorthPop aims to follow the included children until at least 7 years of age, with data being collected at the ages of 18 months, 3 years, and 7 years. With these data, weight trajectories of the offspring cohort will be analyzed in future studies. Since the temporal patterns of the association might be complex and change as the offspring grows, we will also investigate the potential epigenetic mediation of BMI at different ages of the child.

## Conclusions

We present new insights suggesting epigenetic factors as mediators of associations between maternal lifestyle and birthweight in this predominantly Northern European population. Our top findings include identification of eight CpG sites that appear to mediate associations between maternal characteristics (GWG and pregnancy BMI) and children’s birth weight. The most notable methylation site was cg19242268 in *TCEA2*, as DNA methylation of this site was involved in mediation between both characteristics and birth weight. Cord blood DNA methylation surrounding this gene has also previously been implicated as a marker of birth weight [34]. However, most importantly, we believe that our results may increase the general understanding of intergenerational inheritance of obesity and highlights the importance of adhering to healthy lifestyle throughout the life span, which could benefit potentially transcending generations. Future studies are warranted to validate and elucidate the functional mechanisms.

## Supplementary Information

Additional file1 (DOCX 2369 KB)

## Data Availability

Data described in the manuscript, will be made available upon reasonable request pending valid ethical approval as well as approval by the NorthPop steering committee.
